# Supplementary material for: Toward stable replication of genomic information in pools of RNA molecules
Source: eLife. 2025 Oct 23;14:RP104043. doi: 10.7554/eLife.104043 (PMC12549019; doi:10.7554/eLife.104043)
Supplement: Supplementary file 1. [file elife-104043-supp1.pdf]

Supplementary File 1. Genomes sampled via the Metropolis-Hastings algorithm with the motif entropy replacing the energy function. The table summarizes the sequence of the genome, its characteristic length scales L_E and L_U, as well as the motif entropy on all length scales of interest. The keyword "bias" is used to distinguish two different sampling procedures: Weakly biased genomes are designed to obey the desired length scales L_E and L_U while retaining a close-to-uniform motif distribution for subsequences of length $L_E < L < L_U$, whereas the motif distribution is far from uniform for strongly biased genomes.

L_G	L_E	L_U	bias	entropy S	genome	L_G	L_E	L_U	bias	entropy S	genome
64	3	4	none	$S(1) = 1.0$<br>$S(2) = 2.0$<br>$S(3) = 3.0$<br>$S(\geq 4) = 3.5$	CUUUCGUGCCAUCUAC<br>GCCCCUCGGUCUCACU<br>GAACAGGUUACUUAUU<br>GGAUUUGUCCGCUAUG	64	2	4	weak	$S(1) = 1.0$<br>$S(2) = 2.0$<br>$S(3) = 2.961$<br>$S(\geq 4) = 3.5$	UUAGGGUAGUAAUGGC<br>GACUUUCUCACGAGCA<br>CCUGUGUAUUCAUCUU<br>GCCGUCCCAGCGGAUU
64	3	6	weak	$S(1) = 1.0$<br>$S(2) = 2.0$<br>$S(3) = 3.0$<br>$S(4) = 3.469$<br>$S(5) = 3.484$<br>$S(\geq 6) = 3.5$	ACUGCUACAUAAUCUC<br>ACACCCUGGAACGAAA<br>GCGAGUAUCCGUCUUG<br>CCGCCAAUGACCCUAA	64	2	6	weak	$S(1) = 1.0$<br>$S(2) = 2.0$<br>$S(3) = 2.961$<br>$S(4) = 3.469$<br>$S(5) = 3.484$<br>$S(\geq 6) = 3.5$	GCUGUCUCCUCUAUUC<br>ACGAUAAAGGGAAAUG<br>GUAAGAUGCCGCCACC<br>GUUGUAGCGUUGACCU
64	3	8	weak	$S(1) = 1.0$<br>$S(2) = 2.0$<br>$S(3) = 3.0$<br>$S(4) = 3.438$<br>$S(5) = 3.453$<br>$S(6) = 3.469$<br>$S(7) = 3.484$<br>$S(\geq 8) = 3.5$	UUUGAACCCCAUUACA<br>GGCUCGUCCUCCGCCA<br>CCGAUAACUUUGAUGC<br>UAUUCUGCGUAGACAC	64	2	8	weak	$S(1) = 1.0$<br>$S(2) = 2.0$<br>$S(3) = 2.961$<br>$S(4) = 3.438$<br>$S(5) = 3.453$<br>$S(6) = 3.469$<br>$S(7) = 3.484$<br>$S(\geq 8) = 3.5$	AAGAUUGUAUGCCGUC<br>UAAUAGGGUGAAAAGC<br>CUCUGUUACGCUCCUG<br>GUUCACCCGAUGUCGC
64	3	10	weak	$S(1) = 1.0$<br>$S(2) = 2.0$<br>$S(3) = 3.0$<br>$S(4) = 3.406$<br>$S(5) = 3.422$<br>$S(6) = 3.438$<br>$S(7) = 3.453$<br>$S(8) = 3.469$<br>$S(9) = 3.484$<br>$S(\geq 10) = 3.5$	CCUCCGCCAUUACAGG<br>CUAUGCUCGUCCCACU<br>UUGAUUCUGCGUAGAC<br>CGAACACUUUGAUAAC	64	2	10	weak	$S(1) = 1.0$<br>$S(2) = 2.0$<br>$S(3) = 2.961$<br>$S(4) = 3.406$<br>$S(5) = 3.422$<br>$S(6) = 3.438$<br>$S(7) = 3.453$<br>$S(8) = 3.469$<br>$S(9) = 3.484$<br>$S(\geq 10) = 3.5$	CCCAUUGGACUUAGUG<br>GUUGCUCUGUAUGCGG<br>GAACGACCGUGCUCUG<br>UAAAAGGAUAGAUUCG
64	3	4	none	$S(1) = 1.0$<br>$S(2) = 2.0$<br>$S(3) = 3.0$<br>$S(\geq 4) = 3.5$	CUUUCGUGCCAUCUAC<br>GCCCCUCGGUCUCACU<br>GAACAGGUUACUUAUU<br>GGAUUUGUCCGCUAUG	64	2	4	strong	$S(1) = 1.0$<br>$S(2) = 2.0$<br>$S(3) = 2.824$<br>$S(\geq 4) = 3.5$	UACUGUCCGAGCCUCA<br>UUGUUUUGACUCCCGU<br>CGUAGGAUGUAUUAGC<br>GUUGCCCCUGCUGAUU
64	3	6	strong	$S(1) = 1.0$<br>$S(2) = 2.0$<br>$S(3) = 3.0$<br>$S(4) = 3.172$<br>$S(5) = 3.344$<br>$S(\geq 6) = 3.5$	AAACCCUGCUUAUUCA<br>AGCAUCUCCAGGGCGU<br>CGGCGUACACUCCAUC<br>UAGUGUCGGUUUAUUG	64	2	6	strong	$S(1) = 1.0$<br>$S(2) = 2.0$<br>$S(3) = 2.437$<br>$S(4) = 2.863$<br>$S(5) = 3.266$<br>$S(\geq 6) = 3.5$	GCGCUAAAUUAAAUGG<br>UCAUUUUAGUCAGUCC<br>GGUCCAGUCAUGGACU<br>AGCUGACCGCUGGUCC
64	3	8	strong	$S(1) = 1.0$<br>$S(2) = 2.0$<br>$S(3) = 3.0$<br>$S(4) = 3.125$<br>$S(5) = 3.219$<br>$S(6) = 3.313$<br>$S(7) = 3.406$<br>$S(\geq 8) = 3.5$	GGCAUCGCUCUGACAC<br>UAAGGAUGCCCAAUAC<br>UGACACCAAUACCGUU<br>UCCUUAGAGCGAAACG	64	2	8	strong	$S(1) = 1.0$<br>$S(2) = 2.0$<br>$S(3) = 2.358$<br>$S(4) = 2.65$<br>$S(5) = 2.94$<br>$S(6) = 3.176$<br>$S(7) = 3.359$<br>$S(\geq 8) = 3.5$	CUACCAUUCGCUAGGU<br>UCAUUCAUGGUUCGCG<br>CUGAACCUGAACCAUU<br>CAGGUAGCGAACCUAC
64	3	10	strong	$S(1) = 1.0$<br>$S(2) = 2.0$<br>$S(3) = 3.0$<br>$S(4) = 3.078$<br>$S(5) = 3.156$<br>$S(6) = 3.227$<br>$S(7) = 3.297$<br>$S(8) = 3.367$<br>$S(9) = 3.438$<br>$S(\geq 10) = 3.5$	GAGCGUAUCGAAACCC<br>UGCCGGCAUUGUGGAG<br>CGUAUCUUAGUCAGGG<br>UUUCCACAAUGACUAA	64	2	10	strong	$S(1) = 1.0$<br>$S(2) = 2.0$<br>$S(3) = 2.27$<br>$S(4) = 2.502$<br>$S(5) = 2.72$<br>$S(6) = 2.931$<br>$S(7) = 3.136$<br>$S(8) = 3.314$<br>$S(9) = 3.438$<br>$S(\geq 10) = 3.5$	UGACAGCUGUUUCCGG<br>AACCGGUUUCCGGAUA<br>UAUAUACAGCUGUUCA<br>GCUGUUCCGGUUCAGC
